# Implementierungsforschung – effektive Umsetzung evidenzbasierter Interventionen in die Praxis

**DOI:** 10.1007/s00103-025-04088-4

**Published:** 2025-07-04

**Authors:** Charbel El Bcheraoui, Johanna Hanefeld, Lars Schaade

**Affiliations:** 1 Zentrum für Internationalen Gesundheitsschutz, Robert Koch-Institut, Gerichtstraße 27, 13347 Berlin, Deutschland; 2 Robert Koch Institute, Nordufer 20, 13353 Berlin, Deutschland

Der Nachweis der Wirksamkeit gesundheitsbezogener Maßnahmen führt weder automatisch zu deren breiter Umsetzung noch garantiert er den gewünschten Effekt auf Bevölkerungsebene. Gerade in Ländern mit niedrigem und mittlerem Einkommen besteht eine erhebliche Diskrepanz zwischen der Verfügbarkeit wirksamer Interventionen für die Bevölkerungsgesundheit und ihrer tatsächlichen Umsetzung, Skalierung und Effektivität. Verantwortlich hierfür ist eine Vielzahl an Faktoren. Hierzu gehört insbesondere die unzureichende Anpassung von Interventionen an lokale Kontextbedingungen. Zudem lässt die Wirksamkeit von Interventionen häufig nach bzw. verändert sich, wenn Interventionen aus kontrollierten Studienbedingungen in die Anwendung überführt werden – ein Phänomen, das als „Spannungsabfall“ (englisch Voltage Drop) bezeichnet wird.

Probleme in der Umsetzung von evidenzbasierten Interventionen haben auch hohe Relevanz für Public Health in Deutschland: Um das Potenzial von gesundheitsbezogenen Interventionen voll auszuschöpfen, benötigt Public Health in Deutschland kontextspezifisch angepasste Implementierungsstrategien, die kontinuierlich durch Forschung evaluiert und optimiert werden. Diese sollen verhindern, dass die Wirksamkeit einer Intervention durch Diskrepanzen zwischen geplanten und tatsächlichen Ergebnissen gemindert wird, und zugleich eine möglichst hohe Rentabilität der Investitionen gewährleisten. Die zukünftige Entwicklung gesundheitsbezogener Trends in Deutschland wird maßgeblich davon abhängen, inwieweit präventive Strategien im Gesundheits- und Public-Health-Bereich weiterentwickelt, erfolgreich implementiert und von der Bevölkerung akzeptiert werden.

Die Implementierungswissenschaft (englisch Implementation Science, eng verwandt mit der Begleitforschung) ist eine Forschungsrichtung, die sich mit der effektiven Umsetzung von Interventionen in die Praxis befasst. Anders als die Versorgungsforschung, die sich primär mit Gesundheitsversorgungssystemen und -prozessen und deren Nutzung befasst, untersucht die Implementierungswissenschaft Strategien zur Einführung, Anpassung und Verankerung evidenzbasierter Public-Health- und Gesundheitssystem-Interventionen. Sie untersucht dabei gezielt Strategien zur Anpassung und Anwendung evidenzbasierter Interventionen und betont die Notwendigkeit, Interventionen im Verlauf ihrer Umsetzung kontinuierlich zu optimieren und an die komplexen Bedingungen realer Situationen anzupassen. So unterstützt die Implementierungswissenschaft die Übertragung wissenschaftlicher Evidenz in die Praxis – sowohl in Ländern mit niedrigerem als auch mit hohem Einkommen. Implementierungswissenschaft kann im Bereich Public Health entscheidend dazu beitragen, das Potenzial von gesundheitsbezogenen Interventionen voll auszuschöpfen und deren Effektivität in der Umsetzung zu erhöhen. Obwohl in Deutschland und auf europäischer Ebene der Bedarf an Implementierungsforschung zunehmend anerkannt wird, ist die Implementierungswissenschaft in der Gesundheits- und Public-Health-Forschung in Deutschland bislang wenig etabliert. Unklar ist, ob dies auf die kurze Entwicklungsgeschichte der Disziplin zurückzuführen ist oder auf spezifische Hürden, die einer breiteren Anwendung in Deutschland entgegenstehen.

Dieses Themenheft veranschaulicht die Bedeutung und den Nutzen der Implementierungswissenschaft für die Gesundheits- und Public-Health-Forschung anhand praxisnaher Anwendungsbeispiele. Den Auftakt bietet eine kritische Übersichtsarbeit über den Stand und das Potenzial der Implementierungswissenschaft im deutschen Forschungskontext. Anschließend werden Ansätze und Methoden ihrer Anwendung im Gesundheitsbereich vertieft dargestellt.

Den thematischen Einstieg liefert eine Übersicht von *Weishaar et al.*, in der zunächst eine Definition der Implementierungswissenschaft vorgestellt wird. Anschließend fassen die Autor:innen einige der in der Implementierungswissenschaft häufig verwendete Theorien und Rahmenkonzepte zusammen, mit denen Umsetzungsstrategien, Barrieren, förderliche Faktoren und Outcomes von Interventionen untersucht werden. Durch die Einordnung des Forschungsstandes legt diese Arbeit den Grundstein für eine kontextualisierte Betrachtung in Deutschland und Europa.

Hierauf aufbauend diskutieren *Zscheppang et al.* die Rolle von Partizipation und Co-Kreation in der Implementierungsforschung. Die Autor:innen argumentieren, dass Akzeptanz und Wirksamkeit von Gesundheitsmaßnahmen steigen, wenn diese an reale Bedingungen angepasst sind und Interessensgruppen systematisch einbeziehen. Zur Veranschaulichung stellen die Autor:innen 2 Forschungsbeispiele aus Deutschland vor, die zeigen, wie kontinuierliche Beteiligung, Partizipation und Mitgestaltung von Bürger:innen erfolgreich in Implementierungsansätze integriert werden können.

Einen weiteren Beitrag zur Rolle lokaler Akteure leisten *Delamou et al.* mit ihrer Analyse der kommunalen Gesundheitspolitik in Guinea. Auf Grundlage der „Decisional Space Theory“ zeigen sie, wie wichtig es ist, dass Fachkräfte im öffentlichen Gesundheitsdienst ihre Entscheidungsspielräume kennen und aktiv nutzen, um die Bereitstellung kommunaler Gesundheitsleistungen zu verbessern. *Dos Santos Treichel et al.* nehmen eine andere Governance-Ebene in den Fokus und untersuchen, welche Umsetzungsstrategien in Brasilien eingesetzt werden, um die Beteiligung von Interessengruppen durch Forschungslenkungsausschüsse (Research Steering Committees) zu erhöhen.

Im zweiten Teil des Themenheftes werden konkrete Anwendungsbeispiele der Implementierungsforschung vorgestellt. Den Auftakt bildet ein Beitrag von *Merle et al.* aus den USA, der untersucht, welche kontextuellen Faktoren die Umsetzung von APOL1-Gentests bei der klinischen Bewertung von Lebendnierenspender:innen beeinflussen. Basierend auf dem „Consolidated Framework for Implementation Research“ (CFIR) und auf Basis halbstrukturierter Interviews mit Nephrolog:innen der Northwestern University in Chicago und der Georgetown University in Washington DC stellen die Autor:innen fest, dass die Kompatibilität mit bestehenden klinischen Abläufen und die Unterstützung durch das Klinikmanagement die wichtigsten Faktoren in der Implementierung von entsprechenden Gentests sind. Auch soziale Aspekte wie Ethnizität werden im Artikel thematisiert.

*Chiu et al.* aus Australien zeigen auf, wie ein systemdynamischer Ansatz Programme für nichtübertragbare Krankheiten verbessern kann. Anhand des „RE-AIM-Frameworks“ (Reach, Effectiveness, Adoption, Implementation, Maintenance) untersuchen sie hypothetische Pfade zwischen Reichweite, Nachhaltigkeit und Kosten von Interventionen. Sie unterstreichen die Bedeutung innovativer Strategien, um den Kontakt zur Bevölkerung aufzubauen und aufrechtzuerhalten und so Reichweite und Nachhaltigkeit zu verbessern.

*Pfadenhauer et al.* widmen sich der Umsetzung zweier komplexer Interventionen in Südafrika und Deutschland und arbeiten die Herausforderungen bei der Anwendung von Ansätzen der Implementierungswissenschaft heraus. *Reigl et al.* fokussieren auf die lokale Ebene: Anhand einer Kampagne zur Bekämpfung von Parasiten in 2 Landkreisen in Uganda untersuchen sie die von Gesundheitsbehörden wahrgenommenen Chancen und Herausforderungen bei der Durchführung pharmazeutischer Masseninterventionen.

Über die direkte Gesundheitsversorgung hinaus befassen sich die letzten beiden Artikel mit der Rolle der Implementierungswissenschaft in der Einbeziehung von Gesellschaftsgruppen und der gesundheitlichen Chancengleichheit. *Handley et al.* aus den USA zeigen, wie Methoden der Implementierungswissenschaft genutzt werden können, um typische Herausforderungen in den verschiedenen Phasen der angewandten Gesundheitsforschung zu bewältigen. Anschließend erörtern *Fehlberg et al. *aus Australien, wie die Implementierungswissenschaft zur kulturellen Anpassung von Gesundheitsmaßnahmen und zu gesundheitlicher Gerechtigkeit beitragen kann. Anhand von 5 Ansätzen der Implementierungswissenschaft und dem australischen Genom-Screening-Programm „OurDNA“ entwickeln sie einen Rahmen für die systematische Berichterstattung in der Umsetzung von Anpassungsprozessen.

Die meisten der Beiträge des Themenheftes sind in englischer Sprache und ebenso finden sich englische Fachbegriffe in den deutschsprachigen Artikeln. Auch dies spiegelt die Anfänge der Implementierungswissenschaft im deutschen Forschungskontext wider. Mit dieser Ausgabe möchten wir dazu beitragen, diese Situation zu verändern. Wir danken allen Autor:innen für ihre wertvollen Beiträge und hoffen, dass dieses Themenheft einen Beitrag dazu leistet, Implementierungswissenschaft im Bereich Public Health und in der deutschen Wissenschaft weiter zu verankern.

